# In vivo kinematic analysis of failure cases after nonanatomical anterior cruciate ligament reconstruction: a preliminary study

**DOI:** 10.1186/s43019-024-00254-1

**Published:** 2024-12-30

**Authors:** Tomofumi Kage, Shuji Taketomi, Tetsuya Tomita, Takaharu Yamazaki, Ryota Yamagami, Kenichi Kono, Kohei Kawaguchi, Ryo Murakami, Takahiro Arakawa, Takashi Kobayashi, Hiroshi Inui, Sakae Tanaka

**Affiliations:** 1 Department of Orthopaedic Surgery, Faculty of Medicine, The University of Tokyo, 7-3-1 Hongo, Bunkyo-ku, Tokyo, 113-8655 Japan; 2 Graduate School of Health Sciences, Morinomiya University of Medical Sciences, 1-26-16 Nankokita, Suminoe-ku, Osaka, 559-8611 Japan; 3 Department of Information Systems, Faculty of Engineering, Saitama Institute of Technology, 1690 Fusaiji, Fukaya, Saitama 369-0293 Japan; 4 Department of Orthopaedic Surgery, Saitama Medical Center, Saitama Medical University, 1981 Kamoda, Kawagoe City, Saitama 350-8500 Japan

**Keywords:** Kinematics, Anterior cruciate ligament failure, Nonanatomical anterior cruciate ligament reconstruction

## Abstract

**Background:**

Nonanatomical anterior cruciate ligament (ACL) reconstruction occasionally induces ACL failure without an evident injury episode, necessitating revision surgery. Although the in vivo kinematics of ACL deficiency before primary ACL reconstruction are well documented, the kinematics of ACL failure after nonanatomical reconstruction remain unexplored. The aim of this study is to investigate ACL failure kinematics following nonanatomical reconstruction.

**Patients and methods:**

This study enrolled three patients with ACL failure after nonanatomical reconstruction, 20 normal and 16 ACL-deficient knees. The anteroposterior (AP) translation of the medial and lateral femoral condyles and center of the femur and femoral rotation relative to the tibia during squatting were evaluated using a two- to three-dimensional registration technique under fluoroscopy.

**Results:**

Medial AP translation of the nonanatomically reconstructed knee in one patient showed posterior location and abnormal kinematics compared with the ACL-deficient knees. In contrast, the lateral AP position of the nonanatomically reconstructed knees in two patients were more posteriorly located and showed more abnormal kinematics than the ACL-deficient knees. Central AP translation of the nonanatomically reconstructed knees in two patients was located more posteriorly throughout the range of midflexion. Femoral rotation of the nonanatomically reconstructed knees showed abnormal kinematics compared with that of the normal and ACL-deficient knees.

**Conclusions:**

By independently assessing the medial and lateral aspects of the femur, the medial or lateral condyle of the femur of nonanatomically reconstructed knees exhibited a more pronounced abnormality compared with ACL-deficient knees. The femur of the nonanatomically reconstructed knees showed abnormal rotational kinematics. Considering the kinematic aspect, nonanatomical ACL reconstruction should be avoided.

## Background

In patients with ACL injury, anatomic reconstruction of the anterior cruciate ligament (ACL) is critical for restoring knee joint stability and kinematics. When a nonanatomically reconstructed ACL fails, secondary meniscal injuries and/or residual pivot shift are concerns [1]. In such cases, the patient is often unable to return to sports and requires revision surgery, although the initial reconstructive graft is frequently found to be undisrupted at the time of revision surgery [2]. Two clinical situations requiring revision surgery may arise: a situation in which the reconstructed ACL ruptures again because of injury, despite the reconstructed ACL functioning well, and an alternative clinical scenario involving graft failure caused by nonanatomical reconstruction or technical error that gradually manifests without any injury [3]. Previous studies have examined the in vivo kinematics of ACL-deficient knees [4, 5]. The ACL-deficient knees demonstrated femoral posterior translation compared with intact knees. Even in ACL-reconstructed knees, the kinematics may not fully recover and has been associated with poor clinical outcomes in some cases [6]. Conversely, reports of the in vivo kinematics of ACL failure cases after nonanatomical ACL reconstruction are limited, and their kinematic features remain unknown. Elucidating the kinematics of ACL failure after nonanatomical reconstruction is important for planning strategies for subsequent revision surgery. Therefore, we designed this study to describe the kinematics of failure cases after nonanatomical ACL reconstruction.

We present the in vivo kinematics of three cases of ACL failure after nonanatomical ACL reconstruction during squatting. Those patients’ reconstructed knee joints exhibited signs of instability, such as giving way and residual pivot shift, without an evident injury episode after primary reconstruction, and required revision reconstruction surgery. To clarify the difference in ACL failure after nonanatomical reconstruction, we compared normal knees with ACL-deficient knees as a control group. This study aimed to clarify the in vivo kinematics of ACL failure knees after nonanatomical reconstruction and compare it with those of normal and ACL-deficient knees. We hypothesized that the kinematics of ACL failure knees after nonanatomical reconstruction would be more abnormal than that of ACL-deficient knees.

## Patients and methods

### Case descriptions

**Fig. 1:**
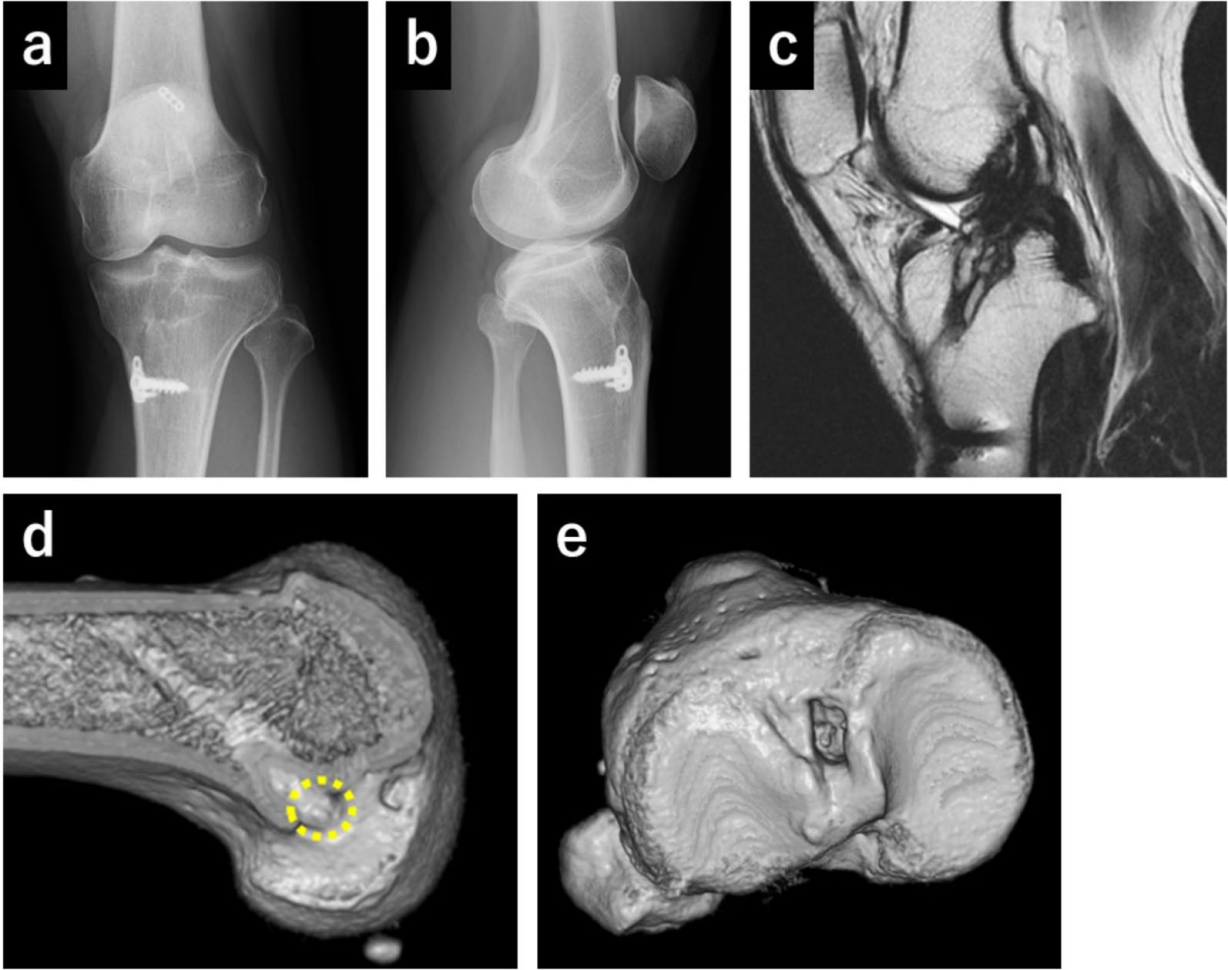
X-ray, magnetic resonance imaging (MRI), and computed tomography (CT) scans in case 1 (**a**–**e**). (**a**) Anteroposterior view of the X-ray. (**b**) Lateral view of the X-ray. (**c**) T1-weighted MRI image of the sagittal plane, showing residual reconstructed anterior cruciate ligament. (**d**) Three-dimensional (3D) CT image of the femoral tunnel position of the left knee. The yellow dot circle shows the aperture of the femoral tunnel. CT shows nonanatomical tunnel position (anterior and distal). (**e**) 3D CT image of the tibial tunnel position

**Fig. 2:**
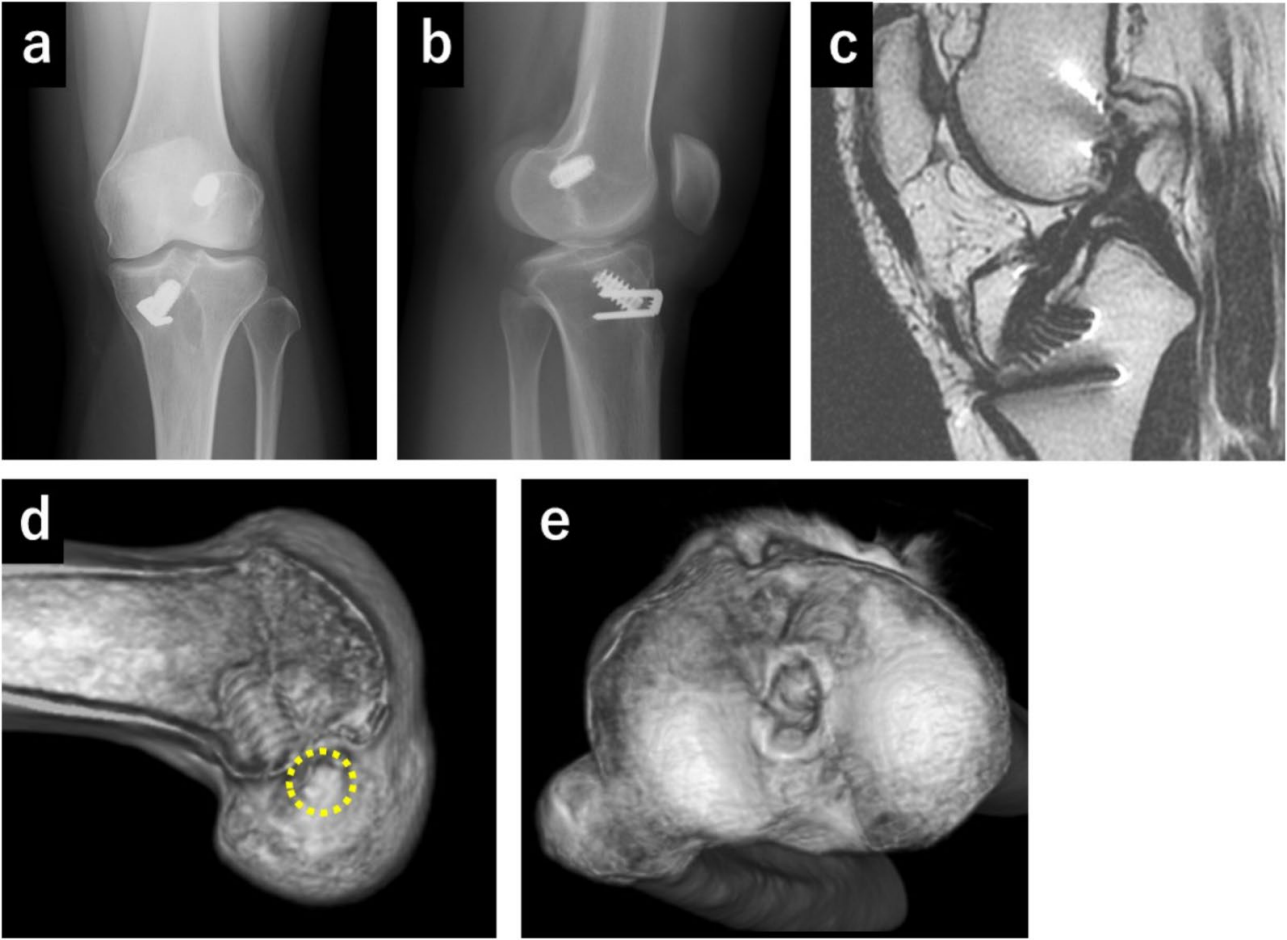
X-ray, magnetic resonance imaging (MRI), and computed tomography (CT) scans in case 2 (**a**–**e**). (**a**) Anteroposterior view of the X-ray. (**b**) Lateral view of the X-ray. (**c**) T1-weighted MRI image of the sagittal plane, showing a partially residual reconstructed anterior cruciate ligament. (**d**) Three-dimensional (3D) CT image of the femoral tunnel position of the left knee. The yellow dot circle shows the aperture of the femoral tunnel. CT shows nonanatomical tunnel position (anterior and distal). (**e**) 3D CT image of the tibial tunnel position, showing a relatively posterior tunnel position

**Fig. 3:**
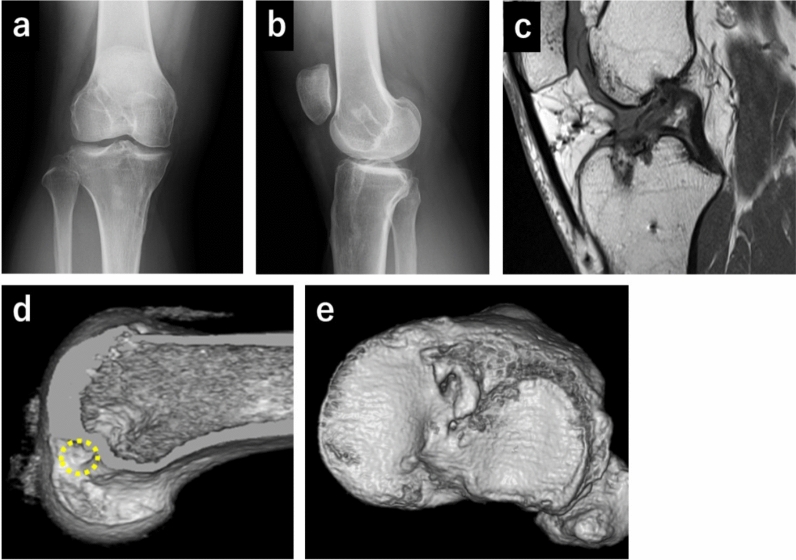
X-ray, magnetic resonance imaging (MRI), and computed tomography (CT) scans in case 3 (**a**–**e**). (**a**) Anteroposterior view of the X-ray. (**b**) Lateral view of the X-ray. (**c**) T1-weighted MRI image of the sagittal plane, showing residual reconstructed anterior cruciate ligament. (**d**) Three-dimensional (3D) CT image of the femoral tunnel position of the right knee. The yellow dot circle shows the aperture of the femoral tunnel. CT shows nonanatomical tunnel position (anterior and distal). (**e**) 3D CT image of the tibial tunnel position

This study was conducted between December 2018 and November 2022 and was approved by our institutional review board [number 2018004P-(8)]. All patients provided written informed consent. Demographic data, including the Knee Injury and Osteoarthritis Outcome Score (KOOS), for the three nonanatomically reconstructed cases, normal knees (*n* = 20), and ACL-deficient knees before primary reconstruction (*n* = 16) are presented in Table 1. The exclusion criteria for all patients were (1) presence of knee osteoarthritis (Kellgren–Lawrence classification ≥ grade II), (2) knees with concomitant ligament injuries or cartilage lesions requiring surgery, and (3) history of knee realignment surgery. The three patients with nonanatomically reconstructed ACLs had undergone primary ACL reconstruction at other hospitals and thereafter exhibited instability due to ACL failure without any evident injury episode. Case 1 (left knee) was a patient who had undergone ACL reconstruction 12 years prior using a hamstring graft and whose knee joint was positive for both the Lachman test (grade 2) and pivot shift test (grade 2). The grade was based on IKDC scores [7]. The range of motion (ROM) was 0–145° of flexion. X-ray, magnetic resonance imaging (MRI), and computed tomography (CT) images are shown in Fig. 1a–e. The MRI scan demonstrated residual reconstructed ACL, while the CT revealed a nonanatomical tunnel position (anterior and distal compared with the anatomical position) [8] on the femoral side. No meniscus injury was observed intraoperatively. Case 2 (left knee) involved a patient whose ACL was reconstructed 19 years prior using a bone–patellar tendon–bone graft, and the knee joint was positive on both the Lachman test (grade 3) and pivot shift test (grade 2) [7]. The ROM was 0–140° of flexion. X-ray, MRI, and CT images are shown in Fig. 2a–e. The MRI showed partially residual reconstructed ACL, and the CT revealed the nonanatomical tunnel as being positioned anteriorly and distally on the femoral side and relatively posteriorly on the tibial side. Intraoperatively, a vertical tear of the midportion of the medial menisci was observed, necessitating suturing. Case 3 (right knee) was a patient who had undergone ACL reconstruction 32 years prior using a iliotibial band; the knee joint was positive on both the Lachman test (grade 3) and pivot shift test (grade 3) [7]. The ROM was 10° of hyperextension to 140° of flexion. X-ray, MRI, and CT images are shown in Fig. 3a–e. The MRI showed residual reconstructed ACL, while the CT revealed a nonanatomical tunnel position located anteriorly and distally on the femoral side. Intraoperatively, a vertical tear of the medial menisci from the middle to posterior portion was observed, necessitating suturing.

**Table 1:** Demographic data

	Case 1	Case 2	Case 3	Normal (*n* = 20)	ACL-deficient (*n* = 16)
Knees (number)	1	1	1	20	16
Gender (male/female)	Male	Female	Female	20/0	13/3
Age (years)	42	35	50	35 ± 2	33 ± 6
Body height (cm)	169	164	158	174 ± 5	171 ± 6
Body weight (kg)	67	62	67	70 ± 8	71 ± 11
BMI (kg/m^2^)	23.5	23.1	26.8	23.0 ± 1.3	24.1 ± 2.9
KOOS symptoms	64	93	71	N/A	84 ± 16
KOOS pain	75	97	83	N/A	85 ± 10
KOOS ADL	63	99	87	N/A	93 ± 6
KOOS sports	35	85	50	N/A	54 ± 28
KOOS QOL	19	81	69	N/A	48 ± 24

Data are shown as the mean ± standard deviation

*ACL* anterior cruciate ligament, *BMI* body mass index, *KOOS* Knee Injury and Osteoarthritis Outcome Score, *ADL* activities of daily living, *QOL* quality of life, *N/A* not applicable

### Kinematic analysis

**Fig. 4:**
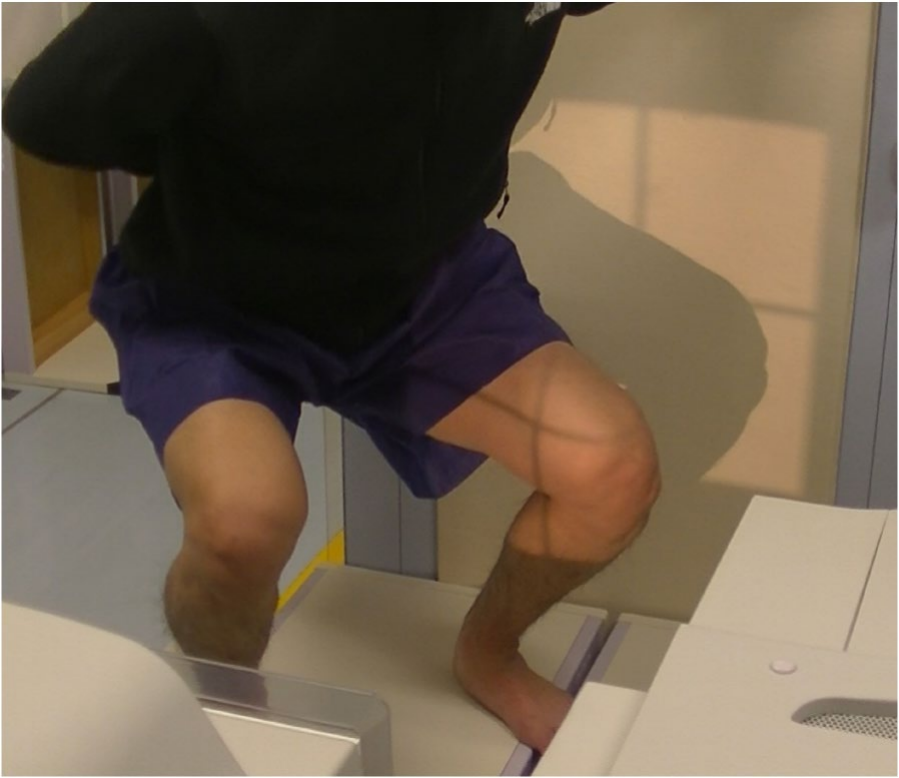
Picture of a patient undergoing kinematic analysis

For the in vivo kinematic evaluation, each participant was instructed to squat while undergoing single-view fluoroscopy in the sagittal plane (Fig. 4), as described previously [5]. A two-dimensional to three-dimensional (2D/3D) registration technique consisting of a contour-based algorithm was used to estimate the spatial position and orientation of the femur and tibia [9]. The relative motion estimation accuracy between the 3D bone models was ≤ 1° for rotation and ≤ 1 mm for translation [10]. A local coordinate system (*x*-, *y*-, and *z*-axis) was devised for the femur and tibia, as per previous studies [11].

**Fig. 5:**
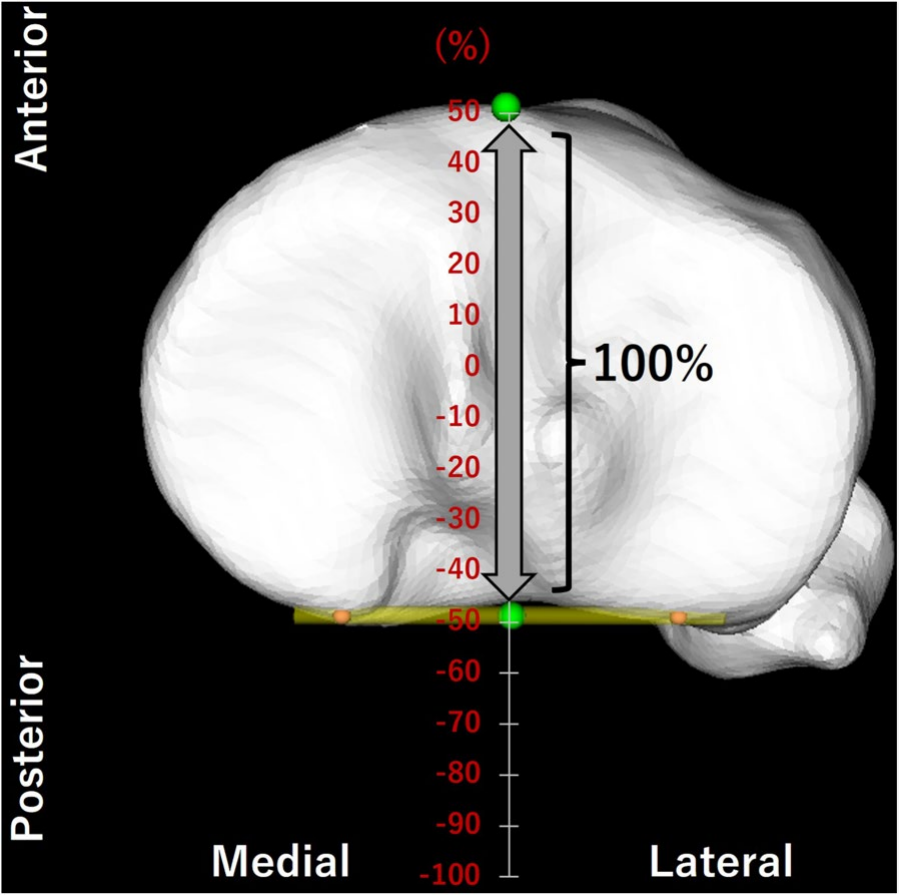
Anteroposterior (AP) dimension of the tibia. The AP translation was calculated as a percentage in relation to the proximal AP dimension of the tibia. The proximal AP dimension of the tibia (gray arrow) was measured as the distance between the most anterior cortical margin and the midpoint (green spheres) of the transverse line connecting the most posterior points of the medial and lateral cortical margins (orange spheres)

Kinematic parameter variables included the anteroposterior (AP) translation of the medial sulcus, lateral epicondyle, and center of the femur, and the rotational angle of the femur relative to the tibia. Femoral rotational angles were calculated using the conventional joint rotation method [12]. AP translation was calculated as a percentage relative to the proximal AP tibial dimension (Fig. 5) [10]. The presence of the femur anterior to the tibia was positive for AP translation. Femoral external rotation relative to the tibia was denoted with positive values. All data are expressed as mean ± standard deviation.

## Results

**Fig. 6:**
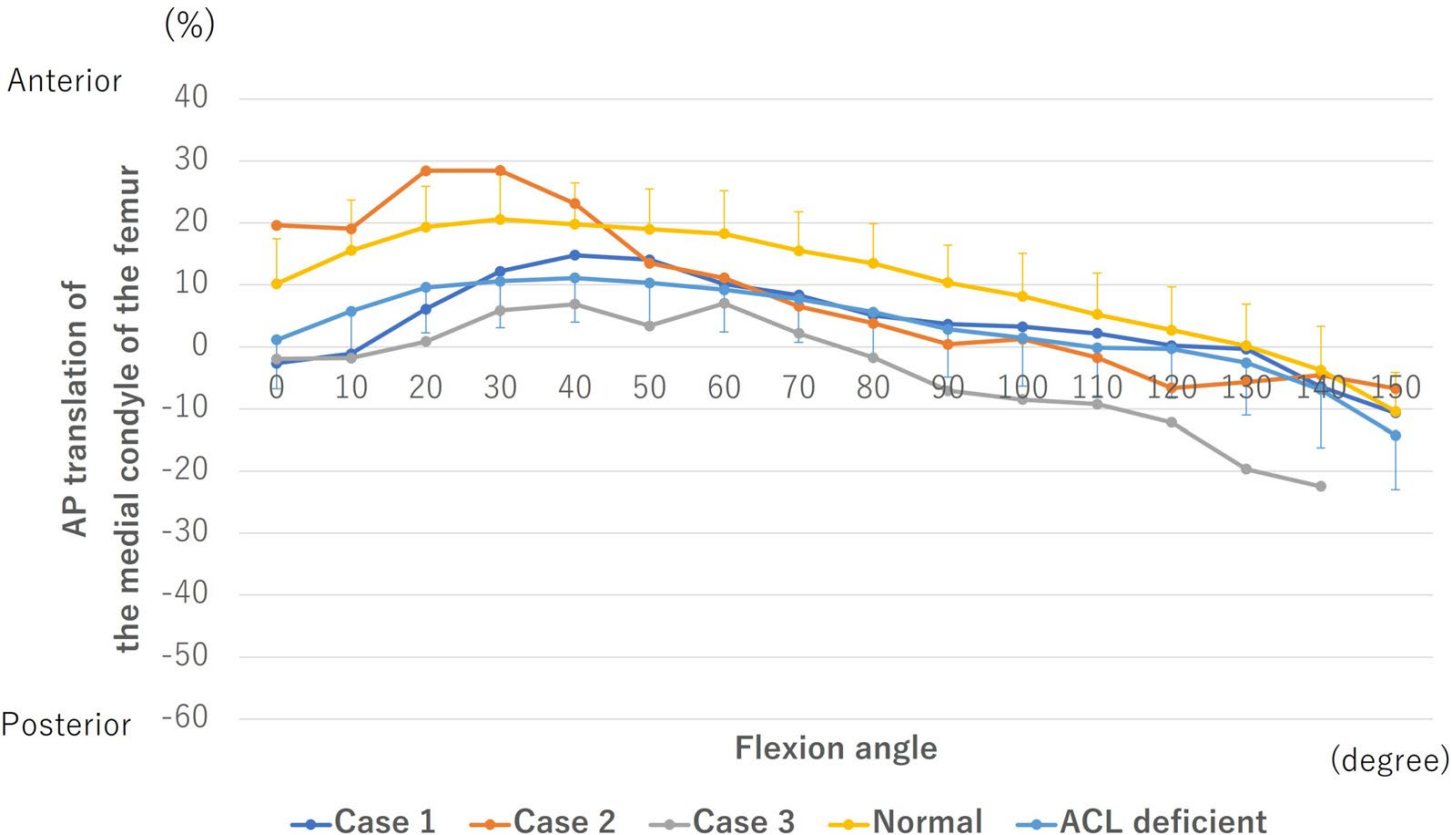
Anteroposterior (AP) translation of the medial condyle of the femur. The anterior or posterior positions with regard to the axis of the tibia are denoted by positive or negative values, respectively. AP translation was calculated as the percent relative to the proximal AP dimension of the tibia. ACL, anterior cruciate ligament

In all knees, the medial condyle of the femur translated anteriorly from 0° to the early flexion angle and then posteriorly (Fig. 6). In case 1, the kinematics of the medial condyle of the femur showed a difference of only 7% compared with those of the ACL-deficient knees. In case 2, the kinematics of the medial condyle of the femur showed a difference of only 7% compared with those of ACL-deficient knees beyond 50° of flexion. In case 3, the medial condyle of the femur was more posteriorly located than that of ACL-deficient knees at all flexion angles.

**Fig. 7:**
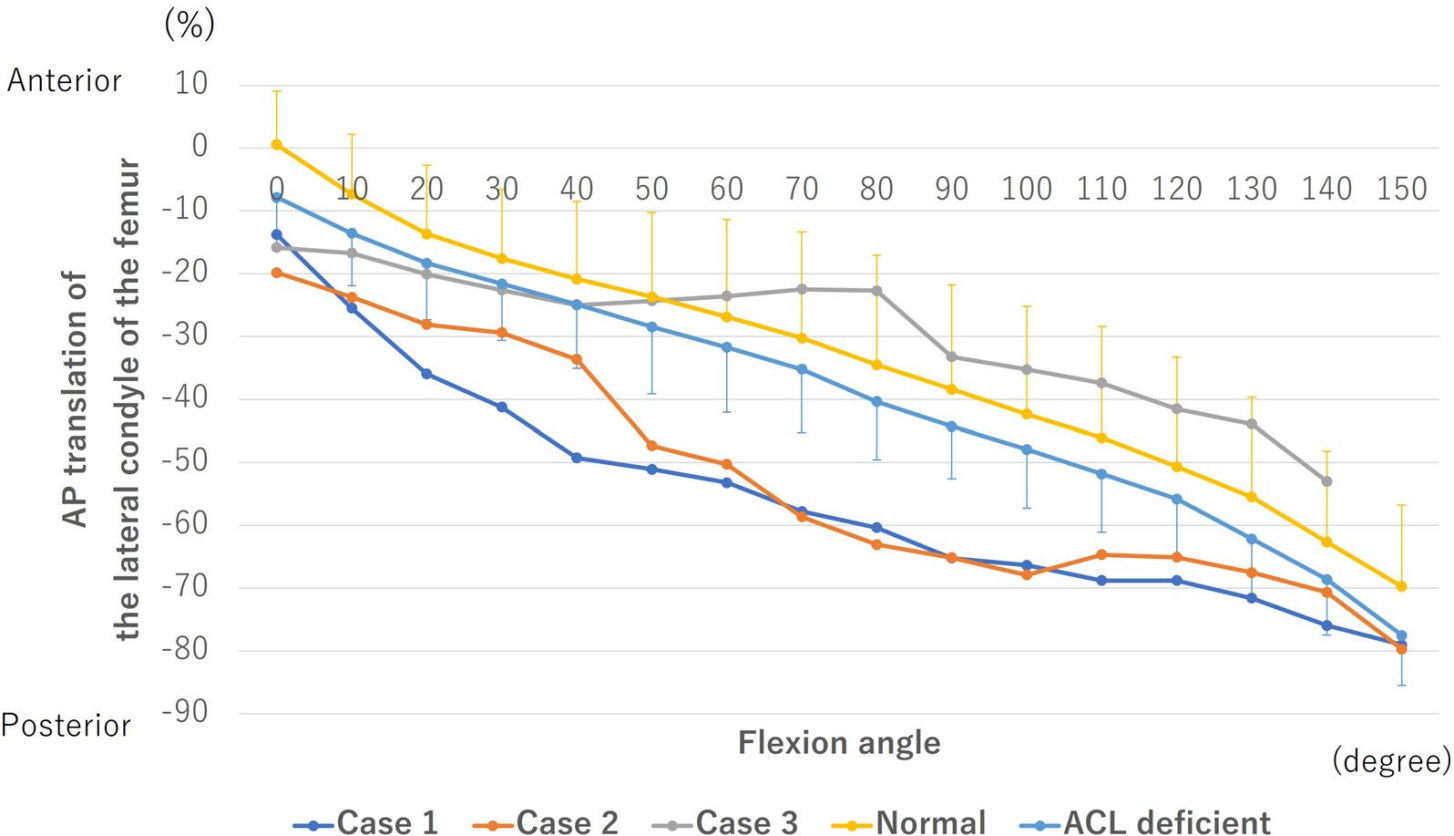
Anteroposterior (AP) translation of the lateral condyle of the femur. The anterior or posterior positions with regard to the axis of the tibia were denoted by positive or negative values, respectively. AP translation was calculated as the percent relative to the proximal AP dimension of the tibia. ACL, anterior cruciate ligament

The lateral condyle of the femur translated posteriorly from 0° to 150° of flexion in all knees (Fig. 7). In cases 1 and 2, the lateral condyle of the femur was posteriorly located compared with the normal and ACL-deficient knees at all flexion angles, in which the difference was up to 30%, especially in the midflexion ranges. In case 3, the difference of the lateral condyle of the femur was 8% compared with the ACL-deficient knees from 0° to 40° of flexion, while the difference of the lateral condyle of the femur was 12% compared with the normal knees from 50° to 140° of flexion.

**Fig. 8:**
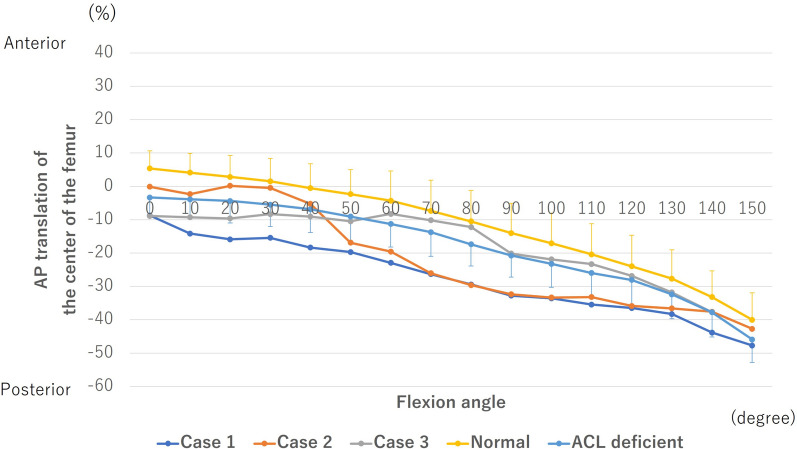
Anteroposterior (AP) translation of the center of the femur. The anterior or posterior positions with regard to the axis of the tibia are denoted by positive or negative values, respectively. AP translation was calculated as the percent relative to the proximal AP dimension of the tibia. ACL, anterior cruciate ligament

The center of the femur translated posteriorly from 0° to 150° of flexion in all knees (Fig. 8). In case 1, the center of the femur was located posteriorly compared with the normal and ACL-deficient knees at all flexion angles. In case 2, the center of the femur was located posteriorly compared with the normal and ACL-deficient knees during midflexion ranges. In case 3, the difference of the center of the femur was 6% compared with the ACL-deficient knees at all flexion angles.

**Fig. 9:**
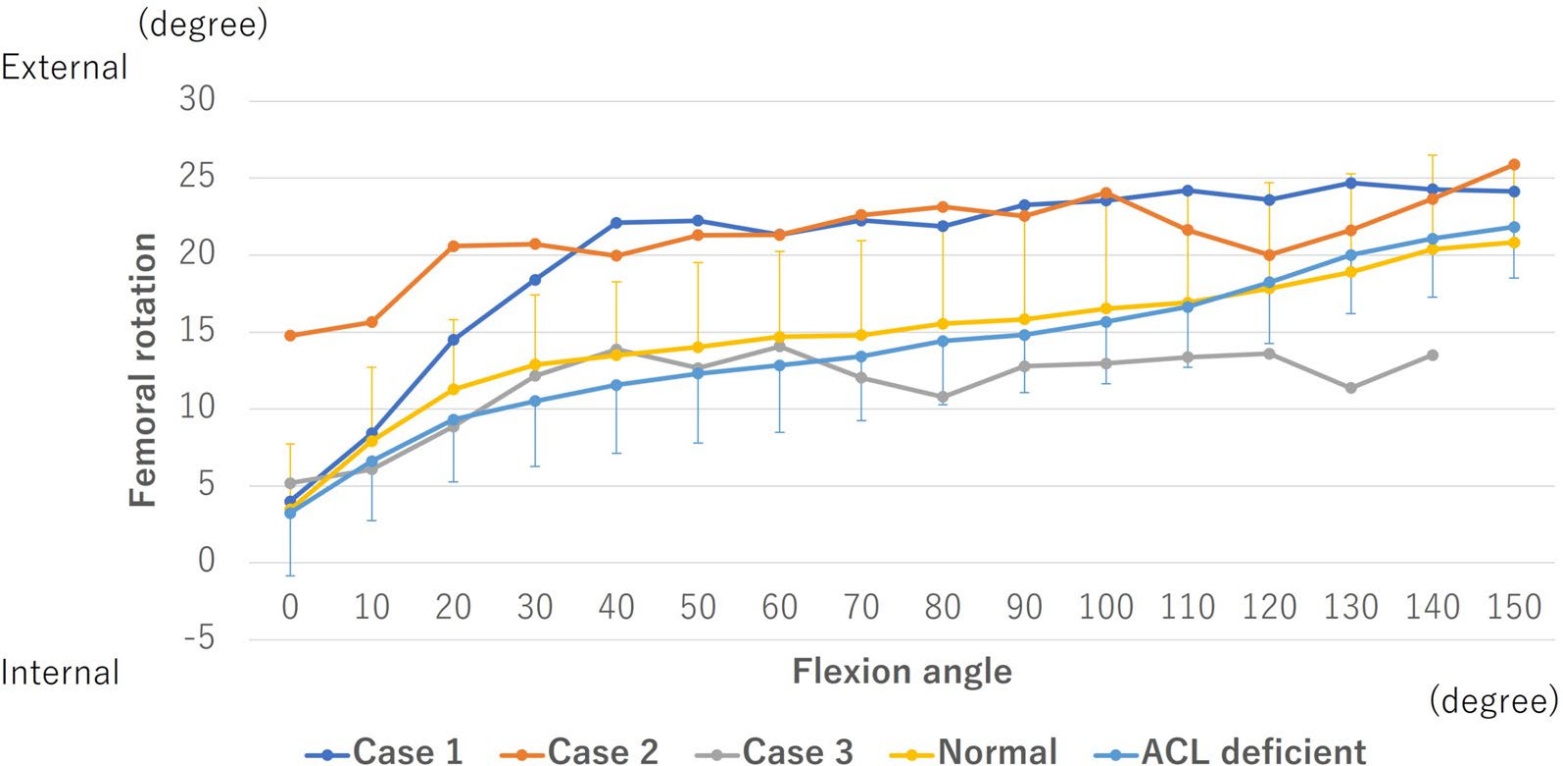
Rotation of the femur. The external rotation of the femur relative to the tibia is denoted by positive values. ACL, anterior cruciate ligament

In normal and ACL-deficient knees, the femur showed approximately 10° external rotation from 0° of flexion to the midflexion range, followed by approximately 5° external rotation up to 150° of flexion (Fig. 9). In contrast, in case 1, the femur showed 18° external rotation from 0° to 40° of flexion and then 2° external rotation up to 150° of flexion. Furthermore, as the patient’s knee joint was flexed in case 2, the femur exhibited approximately 10° external rotation relative to the tibia, beginning at 15° of external rotation. In case 3, the femur showed 9° external rotation from 0° to 40° of flexion, and thereafter, apparent rotation was not observed.

## Discussion

Although the number of cases was limited, the most important finding of this study was that, in nonanatomically reconstructed knees with ACL failure, the medial or lateral condyle of the femur was situated more posteriorly than in ACL-deficient knees. Therefore, knees with ACL failure requiring revision reconstruction surgery may show more abnormal kinematics on the medial or lateral femoral condyle than ACL-deficient knees.

Previous studies have reported the kinematics of nonanatomically reconstructed knees [13, 14]. Abebe et al. reported that the nonanatomical graft placement group, in which the femoral tunnel was anteriorly placed compared with the anatomical group, showed posterior femoral translation during quasistatic lunge. Our study findings match those of Abebe et al. The center of the patient’s femur in case 1 was located posteriorly throughout all flexion ranges, and that in case 2 was located posteriorly during the midflexion range, compared with the normal and ACL-deficient knees. Femoral posterior translation observed in nonanatomically reconstructed knees was attributed to vertical graft orientation. Various studies have reported vertical graft orientation due to the nonanatomical tunnel position [2, 15]. Furthermore, the vertical graft in the sagittal plane requires higher forces to resist the same anterior shear force [16]. Therefore, anterior tibial restraint may be ineffective in nonanatomically reconstructed knees, which explains the posterior location of the femur observed in this study.

**Fig. 10:**
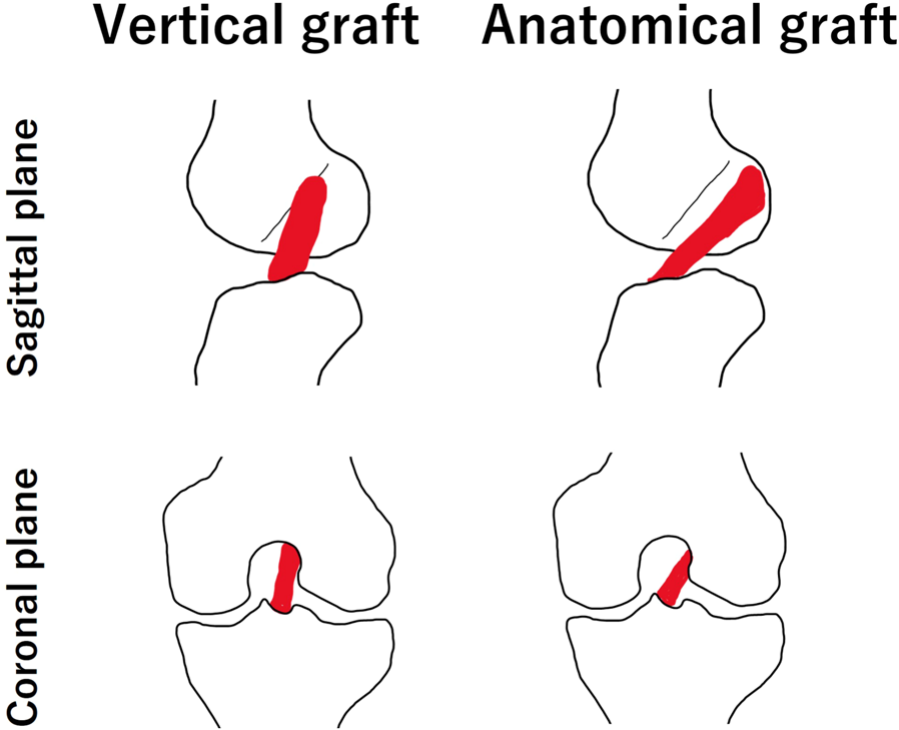
Schema of the vertical and anatomical graft. The running route of the vertical graft is vertical in the sagittal and coronal planes. In contrast, the running route of the anatomical graft is more horizontal in the sagittal and coronal planes compared with the vertical graft

Regarding AP translation, previous studies evaluated only the relationship between the femoral and tibial centers [13, 14]. The medial and lateral aspects have not been assessed separately. The kinematic analysis method utilized enabled us to independently examine the medial and lateral condyles of the femur as well as the center. In case 2, the AP translation of the femur center was midway between that of the normal and ACL-deficient knees at the early flexion angles; however, the lateral condyle of the femur was located posteriorly compared with the ACL-deficient knees at the early flexion angles. The kinematic abnormalities of the lateral femoral condyle were distinctly elucidated using our method. The nonanatomical tunnel position of the vertical graft may explain the aberrant lateral kinematics. Anatomically reconstructed grafts run more horizontally in the sagittal and coronal planes than vertical grafts (Fig. 10). Although the anatomical graft can physiologically retain the lateral femur, the vertical graft cannot. Thus, an abnormal posterior location of the lateral femur may be observed. On another front, in case 3, the medial condyle of the femur was located posteriorly compared with the ACL-deficient knees. One possible reason why the medial condyle of the femur was located posteriorly in case 3 may be attributed to a more vertical graft and severe instability. In fact, the femoral tunnel position in case 3 (Fig. 3d) was more anterior and distal than that of case 1 (Fig. 1d) and case 2 (Fig. 2d). Furthermore, the grade of the Lachman test and the pivot shift test in case 3 was greater than that of case 1 or case 2, showing severe instability. A previous study reported ACL-deficiency-induced posterior location of the femur in the medial compartment [5]. Therefore, the posterior location of the medial condyle of the femur may be induced by severe ACL deficiency. Additionally, meniscal injury, including posterior portion of the menisci in case 3, might be associated with the posterior location of the medial femur. Biomechanically, we discovered an ACL functional deficiency in nonanatomically reconstructed knees.

In terms of femoral rotation, the nonanatomically reconstructed knees in case 1 and case 2 showed external rotation compared with the normal and ACL-deficient knees. The findings of this study are consistent with those of previous studies [13, 14]. These studies reported that the femoral anterior graft placement and nonanatomical graft geometry enhanced femoral external rotation in comparison with anatomically reconstructed knees. By contrast, in case 3, apparent femoral rotation was not observed beyond 40° of flexion, which was different from the normal and ACL-deficient knees. A possible reason for the abnormal rotational kinematics observed in this study may be the vertical graft [2]. The nonanatomical vertical graft was reported to be vertical in both the sagittal and coronal planes compared with the native ACL [15], thus the center of rotation of the femur may be abnormally changed. Therefore, abnormal rotation may have been observed in this study. Our results indicate that knees with ACL failure that require revision surgery could also have abnormal rotational kinematics.

In previous reports, meniscal injuries or osteoarthritic changes after ACL reconstruction have been reported [17]. The abnormal kinematics reported in this study may have caused meniscal injuries or osteoarthritic changes after ACL reconstruction. There are two strong points of our study. First, we evaluated the medial and lateral condyles of the femur separately from the center, thereby elucidating the more abnormal kinematics of the medial or lateral femoral condyle. In our opinion, this is the first report describing the in vivo kinematics of nonanatomically reconstructed knees by separately evaluating the medial and lateral femoral condyles. Second, as a control group, we included ACL-deficient knees before reconstruction; thus, we observed that the kinematics of nonanatomically reconstructed knees was inferior to those of the ACL-deficient knees. However, this study has certain limitations. First, this report included only three nonanatomically reconstructed knees; therefore, the sample size was too small to generalize ACL failure kinematics features after nonanatomical reconstruction. However, there are relatively few cases of ACL failure requiring revision surgery after nonanatomical reconstruction. Therefore, we report this work as a preliminary study. Future studies involving a significant number of knees are required. Second, the knee conditions of the three patients were not similar, including the type of graft, the location of the femoral and tibial tunnels, and the meniscal conditions. Third, the kinematics of the three cases before nonanatomical ACL reconstruction had not been evaluated. Fourth, none of the patients’ contralateral knee kinematics was evaluated.

## Conclusions

We elucidated the in vivo kinematics of knees with ACL failure after nonanatomical ACL reconstruction that required revision surgery by comparing them with normal and ACL-deficient knees. Although the number of cases was limited, the kinematics of the medial or lateral femoral condyle of the nonanatomically reconstructed knees was more abnormal than those of the ACL-deficient knees. Furthermore, the rotational kinematics of the nonanatomically reconstructed knees was abnormal. Knees with nonanatomical ACL reconstruction may have much worse kinematics than ACL-deficient knees. The findings of this study may be useful for understanding ACL failure in nonanatomically reconstructed knees and may provide new insights into treatment strategies.

## Data Availability

The datasets analyzed during the current study are available from the corresponding author on reasonable request.

## Abbreviations

ACL: Anterior cruciate ligament
AP: Anteroposterior
CT: Computed tomography
KOOS: Knee Injury and Osteoarthritis Outcome Score
MRI: Magnetic resonance imaging
ROM: Range of motion
3D: Three-dimensional
